# Yoda1-activated Piezo1 enhances mitophagy in human gingival fibroblasts to promote maxillofacial wound repair

**DOI:** 10.3389/fphar.2026.1803022

**Published:** 2026-04-21

**Authors:** Yuhan Qi, Que Feng, Yan Wu, Jing Xu, Xin Zhang, Lipeng Jiang, Wenjing Liu, Jing Cao, Zhenbao Zhang, Yanfeng Li

**Affiliations:** 1 Medical School of Chinese PLA, Beijing, China; 2 Department of Stomatology, The Fourth Medical Center, Chinese PLA General Hospital, Beijing, China; 3 Department of Radiation Oncology, The First Affiliated Hospital of Jinzhou Medical University, Jinzhou, China; 4 Department of Stomatology, PLA Rocket Force Characteristic Medical Center, Beijing, China

**Keywords:** lipopolysaccharide, mitochondrial autophagy, Piezo1, wound healing, Yoda1

## Abstract

**Background:**

Delayed healing of maxillofacial soft tissue wounds is closely associated with inffammatory injury and mitochondrial dysfunction in fibroblasts, while effective therapeutic strategies remain limited. This study investigated whether activation of the mechanosensitive ion channel Piezo1 by Yoda1 could promote wound healing.

**Methods:**

An LPS-induced inflammatory model was established in human gingival fibroblasts (HGFs) and treated with Yoda1. Intracellular calcium influx, cell viability, oxidative stress, mitochondrial membrane potential, apoptosis, migration, wound closure, and type I collagen expression were assessed. The involvement of the PINK1/Parkin-mediated mitophagy pathway was analyzed. An *in vivo* inflammatory wound model was used to evaluate therapeutic efficacy.

**Results:**

Yoda1 significantly increased intracellular calcium influx, improved cell viability, reduced oxidative stress, restored mitochondrial membrane potential, and inhibited apoptosis in LPS-treated HGFs. It also enhanced HGFs migration, wound closure, and type I collagen expression *in vitro*. Mechanistically, these effects were associated with activation of the PINK1/Parkin-mediated mitophagy pathway. *In vivo*, Yoda1 accelerated wound closure, accompanied by increased collagen deposition and improved tissue regeneration.

**Conclusion:**

Activation of Piezo1 by Yoda1 can alleviate its inflammatory damage by restoring mitochondrial homeostasis and regulating the cellular functions of HGFs. Piezo1 may be a promising therapeutic target for promoting maxillofacial wound repair.

## 1 Introduction

The skin of the maxillofacial region and the oral mucosa serve as the body’s first line of defense against external physical, chemical, and microbial assaults (Harris-Tryon and Grice, 2022). Their integrity is crucial for maintaining local tissue homeostasis and overall systemic health. Epidemiological data indicate that approximately 1%–2% of the global population is affected by hard-to-heal wounds (Järbrink et al., 2016). In the United States alone, over 6.5 million individuals suffer from chronic wounds, and the annual global healthcare costs related to wound management have exceeded hundreds of billions of dollars (Sen, 2025). This burden is particularly pronounced in the maxillofacial area because the soft tissues in this region are continuously exposed to a complex oral microbial environment, significantly increasing the risk of infection and delaying tissue regeneration and repair (Vanlancker et al., 2018). During the healing process of wounds in the maxillofacial skin, mucosa, gingiva, and other soft tissues, fibroblasts are the key cell type responsible for mediating extracellular matrix synthesis and tissue reconstruction (Bainbridge, 2013). Lipopolysaccharides (LPS) derived from Gram-negative bacteria, as critical inflammatory stimuli in the oral microbial environment, can markedly activate the inflammatory response of fibroblasts and induce tissue damage. Numerous studies have shown that this occurs because LPS rapidly induces excessive production of reactive oxygen species (ROS) (Cheng et al., 2015), leading to impaired mitochondrial oxidative phosphorylation and disrupted energy metabolism (Gölz et al., 2014). The resulting imbalance in mitochondrial homeostasis further activates cell death-related signaling pathways, thereby delaying tissue regeneration. Therefore, precise regulation of mitochondrial homeostasis has become a crucial research focus in the field of soft tissue wound repair.

Existing studies have demonstrated that mitophagy, a key mechanism for maintaining mitochondrial homeostasis (Wang et al., 2023), plays a crucial protective role in repairing various tissue injuries (Bullon et al., 2012). It does so by recognizing and clearing damaged mitochondria, preventing the continuous accumulation of ROS, and inhibiting the overactivation of inflammatory pathways (Yang Y. et al., 2025; Zhu et al., 2021). Research by Bi et al. showed that regulating mitophagy in human gingival fibroblasts can suppress the activation of the NLRP3 inflammasome, thereby reducing programmed cell death (Bi et al., 2025). Similarly, Xiao et al. reported that activation of mitophagy enhances the antioxidant capacity of adipose-derived stem cells, leading to reduced apoptosis (Xiao et al., 2025). Therefore, enhancing mitophagy may represent a promising strategy for tissue repair by alleviating cell damage caused by LPS.

When fibroblasts experience impaired oxidative stress function, it severely hinders wound contraction and tissue healing. Literature reports suggest that modulating the mechanical microenvironment of wounds can promote fibroblast migration, differentiation into myofibroblasts, and enhance wound contraction capacity, offering new intervention strategies for soft tissue regeneration (Chen et al., 2025; Xie et al., 2024). Studies in models of myocardial injury (Wang et al., 2021), stem cell homeostasis maintenance (Fang et al., 2025), and bone repair (Zhang et al., 2025) have demonstrated that activating the classic mechanosensitive ion channel Piezo1 can reduce ROS levels and improve cell survival. Yoda1, a specific small-molecule chemical agonist of Piezo1, can continuously activate the Piezo1 signaling pathway without direct mechanical stimulation (Luo et al., 2023), making it an ideal tool for studying the biological effects of mechanosensitive channels (Syeda et al., 2015). However, whether Piezo1 activation can regulate mitochondrial homeostasis in fibroblasts under LPS-induced oxidative stress during maxillofacial soft tissue regeneration and repair remains to be systematically and thoroughly investigated.

The mechanistic link between mechanical environment regulation and mitophagy-mediated protection against LPS-induced cellular damage remains unclear. Elucidating this connection could offer novel mechanobiological strategies to enhance wound regeneration and repair. In this study, we systematically investigated the role of the mechanosensitive ion channel Piezo1 in soft tissue injury. Using an *in vitro* LPS-induced fibroblast injury model and the Piezo1-specific chemical agonist Yoda1 to simulate mechanical signal activation, we found that Piezo1 activation significantly improved fibroblast survival and functional performance within an inflammatory microenvironment. This cytoprotective effect was closely associated with the activation of mitophagy. Furthermore, animal wound healing experiments confirmed the beneficial role of Piezo1 activation in promoting soft tissue repair, suggesting new mechanobiological intervention strategies and potential therapeutic targets for maxillofacial soft tissue wound healing.

## 2 Materials and methods

### 2.1 Cell culture

Human gingival fibroblasts (HGFs) were obtained from the Cell Bank of the Chinese Academy of Sciences (Shanghai, China). Cells were cultured in α-DMEM (Gibco) supplemented with 10% fetal bovine serum (FBS, Gibco) and 1% penicillin-streptomycin solution (100 U/mL penicillin and 100 μg/mL streptomycin). Cell cultures were maintained at 37 °C in a 5% CO_2_ atmosphere. Routine passaging was performed using 0.25% trypsin-EDTA (Gibco). Cells between passages 3 and 8 were used for all experiments.

### 2.2 CCK-8 assay

Cells were seeded at a density of 2 × 10^3^ cells per well in 96-well plates. Cells were treated simultaneously with LPS (1 μg/mL, P. gingivalis LPS) and Yoda1 (Amgicam) at concentrations of 0, 0.1, 0.5, 5, and 10 μM for 24 h. Cytotoxicity and cell proliferation were assessed using the CCK-8 assay. The CCK-8 working solution was prepared by diluting the reagent to 10% (v/v) in complete culture medium, and 100 μL of the solution was added to each well. The plates were incubated at 37 °C for 1 h in the dark, and the absorbance was measured at 450 nm using a microplate reader.

### 2.3 Live/dead staining

HGFs were seeded in 96-well plates at a density of 5 × 10^3^ cells per well for subsequent live/dead staining. Cells were assigned to four experimental groups: Control, receiving no treatment; LPS, treated with 1 μg/mL LPS; Yoda1, co-treated with 1 μg/mL LPS and Yoda1 at 0.1 μM; GsMTx4, co-treated with 1 μg/mL LPS, 0.1 μM Yoda1, and 1 μM GsMTx4, a specific inhibitor of mechanosensitive Piezo1 channels. The abbreviated group names (Control, LPS, Yoda1, and GsMTx4) used in all figures correspond respectively to Control, LPS only, LPS + Yoda1, and LPS + Yoda1+GsMTx4. All treatments were applied for 24 h. Perform live/dead staining using Calcein-AM/PI (Beyotime) reagent. The staining solution consisted of 1 μM Calcein-AM and 1 μM PI diluted in 1 mL PBS. The staining solution was added to the cells and incubated at 37 °C for 30 min in the dark. Capture images using a fluorescence microscope under the FITC and TRITC channels, respectively. Use ImageJ software to count and calculate the number of dead cells (red) in each field of view.

### 2.4 EdU essay

HGFs were seeded into 24-well plates according to the experimental groups. After the cells adhered and were treated for 24 h, EdU working solution (10 μM, Beyotime) was added to the culture medium, and the cells were incubated at 37 °C. The cells were then washed twice with PBS and fixed with 4% paraformaldehyde at room temperature for 15 min. Following fixation, the cells were permeabilized with 0.5% Triton X-100 and washed again with PBS. Prepare the Click reaction solution (containing fluorescent azide, CuSO_4_, and reaction buffer) according to the kit instructions, and incubate it at room temperature for 30 min in the dark to label EdU-incorporated DNA. After the reaction, wash the cells with PBS and counterstain the nuclei with Hoechst dye. Finally, observe and capture images using a fluorescence microscope; EdU-positive cells will exhibit green fluorescence.

### 2.5 Transwell assay

Cell migration ability was assessed using 24-well Transwell inserts. Prior to the experiment, HGFs were cultured in serum-free medium for starvation. After trypsinization, 200 μL of cell suspension containing 4 × 10^4^ cells was added to the upper chamber of each well, while 500 μL of complete medium from different experimental groups was added to the lower chamber. The cells were incubated at 37 °C with 5% CO_2_ for 24 h. After incubation, the cells were fixed with 4% paraformaldehyde for 30 min, stained with 0.1% crystal violet for 30 min, and rinsed with PBS to remove excess dye. Images were randomly captured under an optical microscope, and the number of migrated cells per field of view was quantified.

### 2.6 Scratch assay

HGFs were cultured in 6-well plates until approximately 80% confluence was reached. Prior to the experiment, cells were starved in serum-free medium. A straight scratch was created across the center of each well using a 1000 μL pipette tip held perpendicular to the plate. The wells were gently washed twice with PBS to remove detached cells and debris. Subsequently, culture medium containing the respective treatments was added. Images were captured immediately after scratching (0 h) and again after 36 h at the same location to evaluate cell migration. The remaining wound area at 36 h was measured using ImageJ, and the wound closure rate was calculated relative to the initial wound area at 0 h.

### 2.7 Immunofluorescence

HGFs were seeded at an appropriate density in 24-well plates. After treatment, the cells were washed with PBS and fixed with 4% paraformaldehyde at room temperature for 30 min. The cells were then permeabilized with 0.1% Triton X-100 for 10 min and blocked with 5% BSA for 1 h at room temperature. Subsequently, the cells were incubated with primary antibodies overnight (12–16 h) at 4 °C. The primary antibodies included Yap (13584-1-AP, Proteintech), Collagen I (ab34710, Abcam), and LC3 (T55992, Abmart). After washing with PBS, the cells were incubated with appropriate secondary antibodies for 60 min at room temperature in the dark. The samples were then washed and incubated with phalloidin for 30 min in the dark. Finally, the samples were mounted with DAPI-containing mounting medium and observed under a laser confocal microscope. Fluorescence intensity was analyzed using ImageJ.

### 2.8 Calcium ion detection

HGFs in the respective experimental groups were treated, and intracellular calcium ions were subsequently detected using Fluo-8 AM (Applygen). HGFs cultured in 24-well plates were incubated with 5 μM Fluo-8 AM dye solution prepared in PBS for 30 min in the dark. Following incubation, the Fluo-8 AM solution was removed, and the cells were subsequently incubated with Hoechst 33342 (Beyotime). After washing with PBS to remove excess dye, the cells were observed and imaged using a fluorescence microscope.

### 2.9 Western blotting

HGFs were lysed using RIPA lysis buffer containing protease inhibitors, and protein concentration was determined using a BCA protein assay kit. The protein samples were then mixed with 5× loading buffer (Beyotime) and denatured at 95 °C. Proteins were separated by SDS-PAGE and subsequently transferred onto PVDF membranes. The membranes were blocked at room temperature for 1 h and then incubated with primary antibodies overnight at 4 °C. The primary antibodies included Bax (ab32503, Abcam), Bcl-2 (ab32124, Abcam), Pink1 (23274-1-AP, Proteintech), Parkin (14060-1-AP, Proteintech), β-actin (ab8226, Abcam), and GAPDH (GB15004-100, Servicebio). After washing with TBST, the membranes were incubated with appropriate secondary antibodies at room temperature for 1 h. Protein bands were visualized using an enhanced chemiluminescence (ECL) substrate and quantified using ImageJ software.

### 2.10 Reactive oxygen species (ROS) detection

Intracellular ROS levels were measured using a ROS detection kit (Beyotime) following the manufacturer’s instructions. After treatment, HGFs were incubated with 10 μM 2′,7′-dichlorodihydrofluorescein diacetate (DCFH-DA) in the dark for 1 h. The cells were then washed with PBS and counterstained with Hoechst dye (Beyotime). Fluorescence images were captured using a fluorescence microscope, and fluorescence intensity was quantified to evaluate ROS generation.

### 2.11 Mitochondrial superoxide detection

HGFs were seeded into 48-well plates for the respective treatments. Following the manufacturer’s instructions, the MitoNeoD mitochondrial superoxide probe (10 μmol/L) was prepared and added to the cells, which were then incubated at 37 °C in the dark. After incubation, cell nuclei were stained with Hoechst dye, and fluorescence signals were captured using a fluorescence microscope.

### 2.12 Mitochondrial membrane potential assay

Cells were cultured in 24-well plates and treated with the appropriate drugs. JC-1 working solution (5 μg/mL, Beyotime) was added to the wells, followed by incubation at 37 °C for 30 min. After incubation, the cells were washed with warm PBS and stained with Hoechst 33342 to label the nuclei. Fluorescence images were captured using a fluorescence microscope, and the total intensity of the red and green fluorescence channels for each cell was quantified using ImageJ.

### 2.13 MitoTracker mitochondrial staining

HGFs were seeded onto confocal dishes and allowed to adhere overnight before experimental grouping and treatment. After treatment, 100 nM MitoTracker Red was prepared in serum-free DMEM and incubated with the cells at 37 °C in the dark for 30 min. Following incubation, the cells were washed three times with PBS and then fixed with 4% paraformaldehyde for 15 min. Cell nuclei were counterstained with Hoechst dye. Finally, image acquisition was performed using a laser confocal microscope, and quantitative analysis of the co-localization of mitochondria and LC3 was conducted using ImageJ software.

### 2.14 Transmission electron microscopy

For ultrastructural analysis, cultured fibroblasts were harvested by trypsinization and centrifugation to obtain cell pellets. The pellets were immediately fixed in an electron microscopy fixative for 30 min at room temperature, followed by resin embedding according to standard protocols. Semi-thin sections were first prepared and stained to identify regions of interest. Subsequently, ultrathin sections were cut using an ultramicrotome and collected on copper grids. The sections were then contrasted with heavy metal stains to enhance the visualization of intracellular structures. Finally, the ultrastructural features of cellular organelles were examined and imaged using a transmission electron microscope.

### 2.15 Animal modeling

Male Sprague-Dawley (SD) rats aged 8–10 weeks (weighing 250–300 g) were selected for the study. All animal experiments were approved by the Animal Ethics Committee (Ethics Approval Number: VS2126A00743) and strictly adhered to the Regulations on the Administration of Laboratory Animals. After 1 week of acclimatization, anesthesia was induced by intraperitoneal injection of pentobarbital sodium (40 mg/kg). The dorsal hair was shaved and disinfected with 75% alcohol. Using a sterile punch biopsy tool, a full-thickness skin defect wound with a diameter of 10 mm was created on both sides of the dorsal midline, removing the epidermis, dermis, and subcutaneous tissue. To establish an inflammatory wound model, lipopolysaccharide (LPS; Sigma-Aldrich) was used for local induction. Immediately after wound creation, LPS was delivered at 0.5 mg/kg via intradermal injection around the wound margins, beginning on day 0 and repeated every other day. The animals were randomly assigned to four groups: (1) Control, receiving no treatment; (2) LPS, receiving LPS to induce local inflammation; (3) Yoda1, receiving LPS combined with Yoda1 treatment; and (4) GsMTx4, receiving LPS and Yoda1 together with the Piezo1 inhibitor GsMTx4. All treatments were administered according to the procedures described in the wound model section. For each wound, a total volume of 100 μL of the respective treatment was injected at four points around the wound every other day (25 μL per site). Tissue samples were collected on postoperative day 14 for histological and molecular biological analyses.

### 2.16 Histological analysis

Collected tissues were fixed in 4% paraformaldehyde for 24 h, then dehydrated, embedded in paraffin, and sectioned at a thickness of 5 μm. Hematoxylin and eosin (H&E) and Masson’s trichrome staining were performed and examined under a light microscope. For immunofluorescence staining, sections were deparaffinized, subjected to antigen retrieval, blocked, permeabilized, and incubated with primary antibodies. The primary antibodies used included CD31 (YP-Ab-16897, UpinBio), Piezo1 (A4340, ABclonal), and Ki67 (27309-1-AP, Proteintech). After incubation with secondary antibodies, DAPI staining was performed, and the samples were observed under a fluorescence microscope.

### 2.17 Statistical analysis

All quantitative data are presented as the mean ± standard deviation (SD) from at least three independent experiments (N ≥ 3). Statistical analyses were conducted using GraphPad Prism software (version 9.5). Before performing comparative analyses, data were assessed for homogeneity of variance. For comparisons among multiple groups, one-way analysis of variance (ANOVA) was applied, followed by Tukey’s *post hoc* test for multiple comparisons. A p-value of less than 0.05 was considered statistically significant. *, *p*-value <0.05; **, *p*-value <0.01; ***, *p*-value <0.001.

## 3 Results

### 3.1 Yoda1 regulates fibroblast survival and proliferation

**FIGURE 1:**
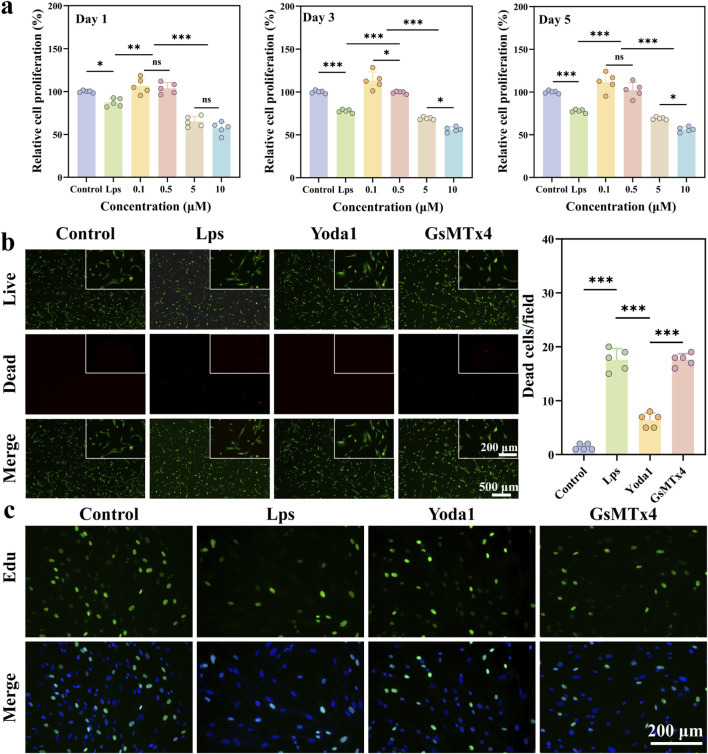
Effects of Yoda1 on the viability and proliferation of human gingival fibroblasts (HGFs) under LPS treatment. **(a)** Cell viability of HGFs treated with varying concentrations of Yoda1 was assessed using the Cell Counting Kit-8 (CCK-8) assay to evaluate cytotoxicity and determine the optimal working concentration (n = 5 per group). **(b)** Representative fluorescence images of Calcein-AM/Propidium Iodide (PI) staining showing live cells (green) and dead cells (red) under different experimental conditions, along with quantitative analysis of dead cells (n = 5 per group). **(c)** EdU assay was performed to assess HGF proliferation, with green fluorescence indicating proliferating cells and nuclei counterstained with Hoechst in blue. Data are presented as mean ± standard deviation from at least three independent experiments (n = 5 per group). Differences between groups were analyzed by one-way ANOVA (*P < 0.05, **P < 0.01, ***P < 0.001).

CCK-8 results demonstrated that Yoda1 exerted a significant bidirectional effect on cell proliferation at varying concentrations (Figure 1a). Compared to the control group, low doses of Yoda1 (0.1 μmol/L and 0.5 μmol/L) significantly increased cell viability, indicating that moderate activation of Piezo1 promotes cell proliferation. Conversely, high doses of Yoda1 (5 μmol/L and 10 μmol/L) significantly inhibited cell proliferation in a dose-dependent manner, with a marked decrease in cell viability, suggesting that excessive activation of Piezo1 may induce toxic effects. Therefore, 0.1 μmol/L Yoda1 was selected as the intervention concentration for subsequent mechanistic experiments, as it maintained cell viability while exhibiting a favorable pro-proliferative effect.

Live/dead staining results further confirmed the effects of different treatments on cell viability. Compared to the control group, the LPS-treated group exhibited an approximately 10-fold increase in the number of dead cells per field of view, showing the highest proportion of cell death (Figure 1b). This indicates that inflammatory stimulation markedly induces cell damage and death. Following Yoda1 intervention in conjunction with LPS treatment, the number of dead cells was reduced by nearly 3-fold. And cell survival was notably improved, demonstrating that activation of Piezo1 can partially alleviate LPS-induced cell damage.

To evaluate the effects of LPS intervention and Piezo1 activation on fibroblast proliferation, we conducted an EdU assay (Figure 1c). The results showed that the proportion of EdU-positive cells was significantly reduced in both the LPS and GsMTx4 groups, indicating inhibited cell proliferation. Further quantification of relative cell proliferation shows that following Yoda1 treatment, the relative cell proliferation increased to approximately 106% relative to the control group. These findings suggest that Yoda1-mediated activation of Piezo1 can reverse the inhibitory effect of LPS on cell proliferation.

### 3.2 Piezo1 activation promotes fibroblast migration and type I collagen production

**FIGURE 2:**
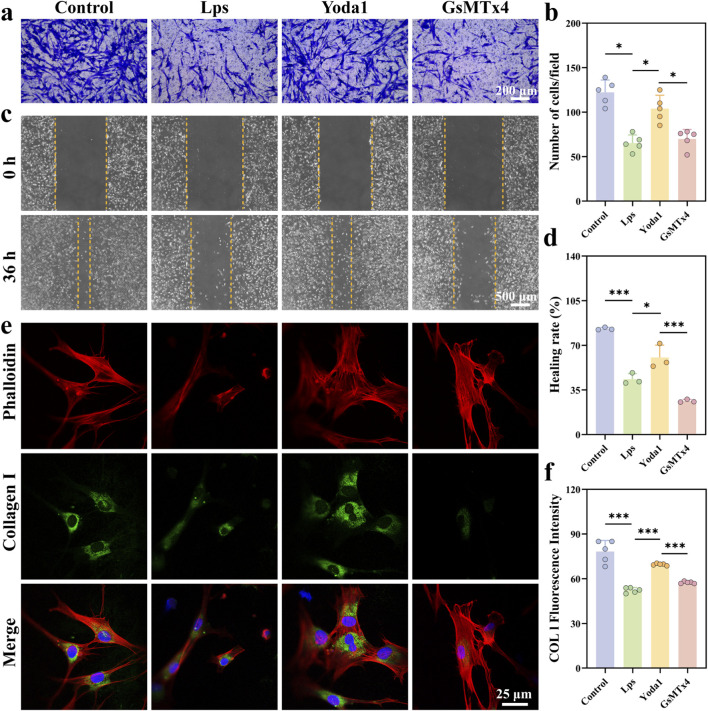
Yoda1 promotes the migration of human gingival fibroblasts (HGFs) and extracellular matrix formation following lipopolysaccharide treatment. **(a,b)** Representative images and quantitative analysis of Transwell migration assays stained with crystal violet under various treatment conditions (n = 5 per group). **(c,d)** Wound healing (scratch) assay illustrating changes in HGFs migration before and 36 h after the scratch, accompanied by quantitative analysis (n = 3 per group). **(e)** Immunofluorescence staining of F-actin (phalloidin, red) and type I collagen (Col I, green) in HGFs under different treatment conditions. Cell nuclei were stained with DAPI (blue). **(f)** Quantitative analysis of type I collagen fluorescence intensity (n = 5 per group). Data are presented as mean ± standard deviation from at least three independent experiments. Differences between groups were analyzed using one-way ANOVA (*P < 0.05, **P < 0.01, ***P < 0.001).

To evaluate the effects of LPS and Yoda1 on the migratory capacity of human gingival fibroblasts (HGFs), a Transwell migration assay was performed. The results demonstrated that the LPS and GsMtx4 groups exhibited the lowest cell migration rates. In contrast, the Yoda1 group showed a significant increase in migratory ability. Notably, Yoda1 partially alleviated the LPS-induced inhibition of HGFs migration (Figures 2a,b). The scratch assay results mirrored this trend, as shown in Figures 2c,d. After 36 h, LPS significantly suppressed HGFs migration (*P* < 0.001). The migration healing area in the Yoda1 group was significantly larger than that in the LPS group (*P* < 0.05). Immunofluorescence co-staining revealed significant differences in cytoskeletal morphology and type I collagen expression among the treatment groups (Figures 2e,f). In the control and Yoda1 groups, fibroblasts exhibited a relatively regular, spread morphology, with F-actin primarily localized along the cell periphery and stress fibers. The fluorescence signal of type I collagen (COL I) was markedly enhanced, predominantly around within the cytoplasm. Conversely, the LPS and GsMtx4 groups displayed disorganized cytoskeletal structures, significantly reduced cell spreading area, and a more contracted morphology. F-actin organization was loosened, accompanied by a diminished COL I fluorescence signal. Yoda1 intervention simultaneously improved abnormal cell morphology and enhanced extracellular matrix synthesis capacity, restoring cytoskeletal tension and mechanical homeostasis.

### 3.3 Piezo1 signal-mediated Ca^2+^ influx, YAP activation, and alterations in cell apoptosis

**FIGURE 3:**
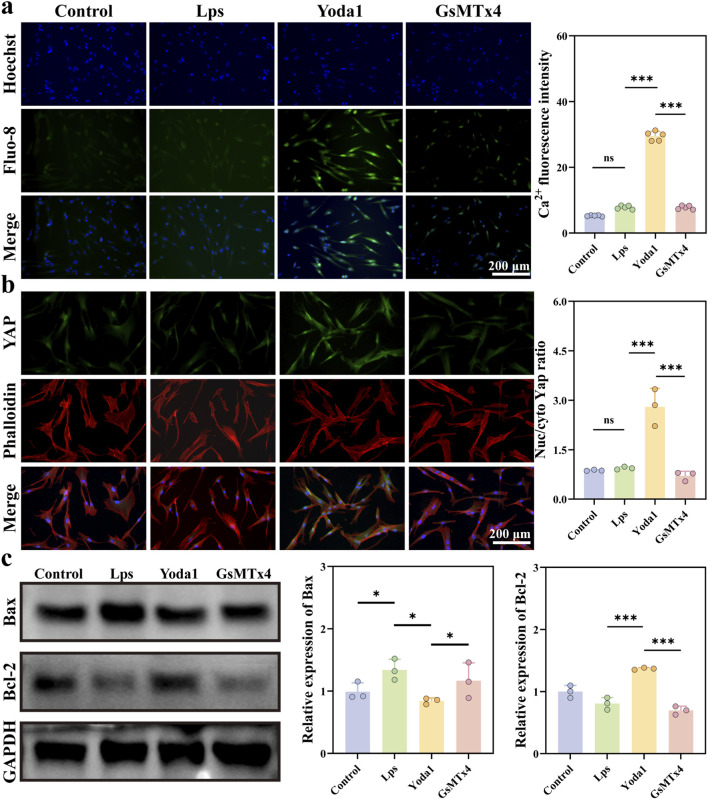
Yoda1 promotes YAP nuclear translocation through calcium ion influx and inhibits cell apoptosis. **(a)** Intracellular calcium ion (Ca^2+^) influx levels in human gingival fibroblasts (HGFs) under various treatment conditions were detected using the Fluo-8 AM fluorescent probe. Representative fluorescence images (green) and quantitative analyses of fluorescence intensity are shown (n = 5 per group), with nuclei counterstained with DAPI (blue). **(b)** Immunofluorescence staining was used to detect the expression, distribution, and quantitative statistics of the YAP transcription factor (green) and cytoskeleton (F-actin, labeled with phalloidin) in HGF under different treatments (n = 3 per group), with nuclei counterstained with DAPI (blue). **(c)** Western blot analysis of apoptosis-related proteins Bax and Bcl-2 expression levels in HGFs under different treatments, with grayscale value quantification (n = 3 per group,all proteins levels are normalized to loading control, GAPDH). Quantitative data are presented as mean ± standard deviation (mean ± SD) from at least three independent experiments. Differences between groups were analyzed by one-way ANOVA (*P < 0.05, **P < 0.01, ***P < 0.001).

To evaluate the effect of Yoda1-mediated activation of Piezo1 on intracellular calcium levels, the Fluo-8 calcium fluorescent probe was used to measure intracellular Ca^2+^ concentrations. As shown in Figure 3a, cells treated with Yoda1 exhibited a significant increase in Fluo-8 fluorescence intensity, indicating a substantial rise in calcium influx. This suggests that Yoda1 effectively induces Ca^2+^ entry, confirming the successful activation of the Piezo1 ion channel. To further assess the impact of Piezo1 activation on fibroblast function, immunofluorescence staining was performed for the transcription factor YAP and the cytoskeleton. As shown in Figure 3b, cells treated with Yoda1 demonstrated enhanced nuclear localization of YAP, whereas cells in the Piezo1 inhibition group showed a significant reduction in nuclear YAP signal. To investigate the cellular consequences of LPS-induced injury, Western blot analysis was conducted to measure the expression levels of apoptosis-related proteins Bcl-2 and Bax. As shown in Figure 3c, compared to the control group, LPS treatment significantly downregulated the anti-apoptotic protein Bcl-2 and markedly upregulated the pro-apoptotic protein Bax, indicating that the LPS-induced inflammatory microenvironment promotes apoptotic signaling in cells. After Yoda1 intervention, the LPS-induced changes described above were significantly reversed, suggesting that Piezo1 activation alleviates LPS-induced cellular apoptosis stress to some extent.

### 3.4 Regulation of reactive oxygen species, mitoneogenesis, and mitochondrial membrane potential by Piezo1

**FIGURE 4:**
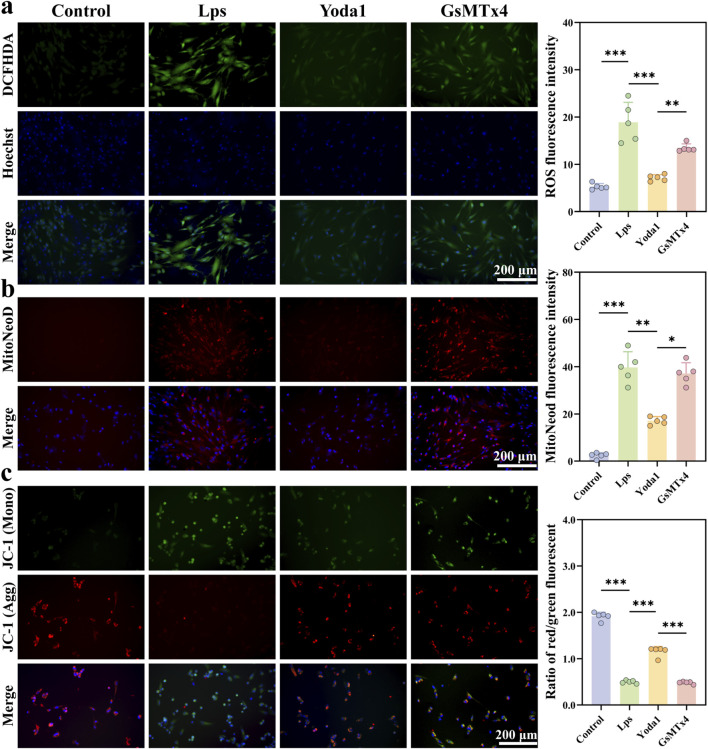
Yoda1 Mitigates LPS-Induced Oxidative Stress and Mitochondrial Dysfunction in Human Gingival Fibroblasts (HGFs). **(a)** Intracellular reactive oxygen species (ROS) levels in HGFs from various treatment groups were detected using the DCFH-DA probe. Representative fluorescence images (green) and quantitative analyses of fluorescence intensity are shown. Cell nuclei were stained with Hoechst (blue) (n = 5 per group). **(b)** Mitochondrial superoxide levels in HGFs from different treatment groups were measured using the mitochondrial superoxide fluorescent probe (MitoNeoD, red). Cell nuclei were stained with Hoechst (blue) (n = 5 per group). **(c)** Mitochondrial membrane potential (ΔΨm) in HGFs from different treatment groups was assessed by JC-1 staining. Representative fluorescence images and quantitative analyses of the red/green fluorescence ratio are presented. Cell nuclei were stained with Hoechst (blue) (n = 5 per group). Quantitative data are expressed as mean ± standard deviation (mean ± SD) from at least three independent experiments. Differences between groups were analyzed by one-way ANOVA (*P < 0.05, **P < 0.01, ***P < 0.001).

Signaling results from the DCFH-DA fluorescent probe assay demonstrated that LPS significantly increased the production of reactive oxygen species (ROS) in human gingival fibroblasts, with fluorescence intensity rising approximately threefold (*P* < 0.001). Compared to the LPS group, Yoda1 markedly reduced intracellular ROS levels (*P* < 0.001), whereas inhibition of Piezo1 led to elevated ROS levels (Figure 4a), suggesting that activation of Piezo1 effectively alleviates oxidative stress. To further localize mitochondrial ROS levels, MitoNeoD fluorescent staining revealed significant differences in mitochondrial status among HGFs across different treatment groups. As shown in Figure 4b, control group cells exhibited only weak MitoNeoD fluorescence signals, indicating that mitochondrial function was relatively stable. After LPS stimulation and GsMTx4 intervention, MitoNeoD fluorescence signals within the cells were significantly enhanced, primarily distributed in the cytoplasm as punctate or clustered aggregates, indicating aggravated mitochondrial damage or abnormalities. In contrast, following Piezo1 activation by Yoda1, MitoNeoD fluorescence signals decreased and were more evenly distributed overall, suggesting that Piezo1 activation effectively mitigates LPS-induced mitochondrial abnormalities.

To evaluate the effect of Yoda1 on mitochondrial function in fibroblasts, JC-1 staining was employed to assess the mitochondrial membrane potential (ΔΨm). As shown in Figure 4c, LPS treatment significantly decreased the JC-1 red/green fluorescence ratio, indicating mitochondrial membrane depolarization and functional impairment. In contrast, treatment with Yoda1 significantly restored the red/green fluorescence ratio, suggesting that Yoda1 partially alleviates LPS-induced mitochondrial dysfunction. These results indicate that Yoda1-mediated activation of Piezo1 helps maintain mitochondrial homeostasis and mitigates mitochondria-related damage signals.

### 3.5 Piezo1 activation induces mitophagy through the PINK1/Parkin pathway

**FIGURE 5:**
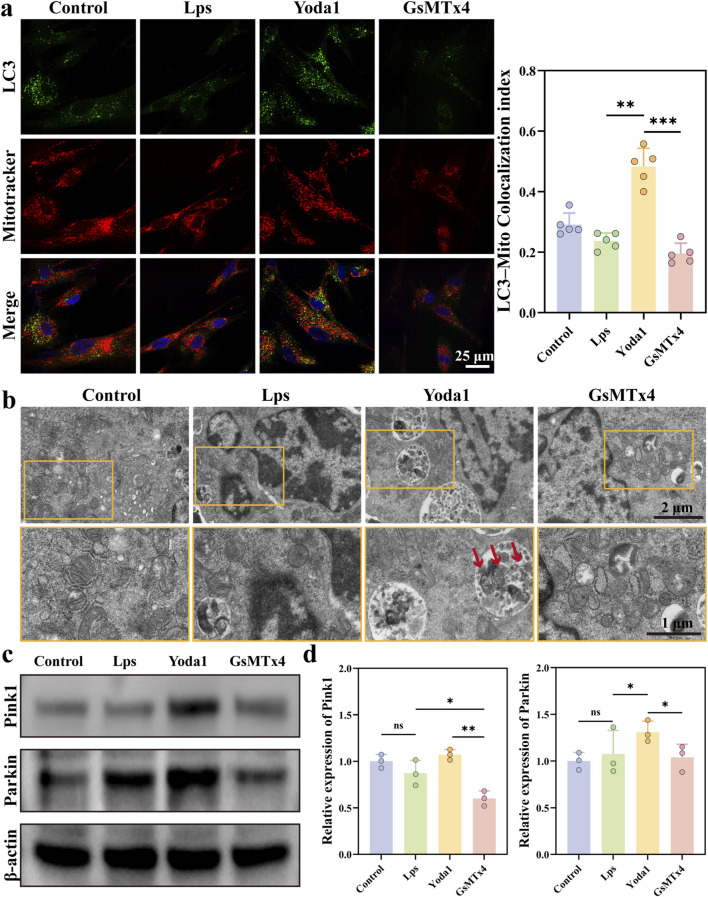
Yoda1 promotes mitophagy through the PINK1/Parkin pathway. **(a)** Representative immunofluorescence images showing co-staining of LC3 (green) and the mitochondrial marker MitoTracker (red) in HGFs under different treatment conditions. Colocalization was quantitatively analyzed using the Pearson correlation coefficient (n = 5 per group). **(b)** Transmission electron microscopy (TEM) images depicting mitochondrial ultrastructure and autophagosome formation in HGFs from various treatment groups. Red arrows indicate instances of mitophagy (n = 3 per group). **(c,d)** Western blot analysis of mitophagy-related proteins PINK1 and Parkin expression levels in HGFs under different treatments, with grayscale quantification (n = 3 per group). Quantitative data are presented as mean ± standard deviation (mean ± SD) from at least three independent experiments. Differences between groups were assessed by one-way ANOVA (*P < 0.05, **P < 0.01, ***P < 0.001).

Immunofluorescence results demonstrated that in the Yoda1 treatment group, the colocalization of LC3 with mitochondria was significantly enhanced, with the Pearson correlation coefficient reaching its highest level (Figure 5a). This indicates that Piezo1 activation markedly promotes mitophagy. In contrast, following the addition of the Piezo1 inhibitor GsMTx4, the colocalization of LC3 with mitochondria was noticeably reduced, and mitophagy levels significantly decreased, suggesting that Yoda1-induced mitophagy depends on the functional activation of Piezo1. Transmission electron microscopy further validated these findings at the ultrastructural level (Figure 5b). Mitochondria in control group cells exhibited intact structures, whereas in the LPS group, some mitochondria showed swelling and blurred cristae. Compared to these, the Yoda1 treatment group displayed more autophagosomes and autolysosomes containing mitochondria, with a significant increase in mitophagy, indicating enhanced cellular autophagic activity. However, after GsMTx4 intervention, damaged mitochondrial structures accumulated again.

To further investigate the molecular mechanism of mitophagy, Western blot analysis was performed on proteins related to the PINK1/Parkin pathway (Figure 5c). The results showed that, compared to the control and LPS groups, the expression levels of PINK1 and Parkin proteins were significantly upregulated in the Yoda1 intervention group. This suggests that Piezo1 activation promotes the initiation of mitophagy through the classical PINK1/Parkin signaling pathway. In contrast, following GsMTx4 intervention, the expression of PINK1 and Parkin proteins was significantly reduced, consistent with the observed decrease in mitophagy levels (Figure 5d). These findings further confirm that Yoda1 primarily exerts its effects by activating Piezo1 and regulating the PINK1/Parkin signaling pathway.

### 3.6 Yoda1 promotes wound closure and re-epithelialization in rat skin

**FIGURE 6:**
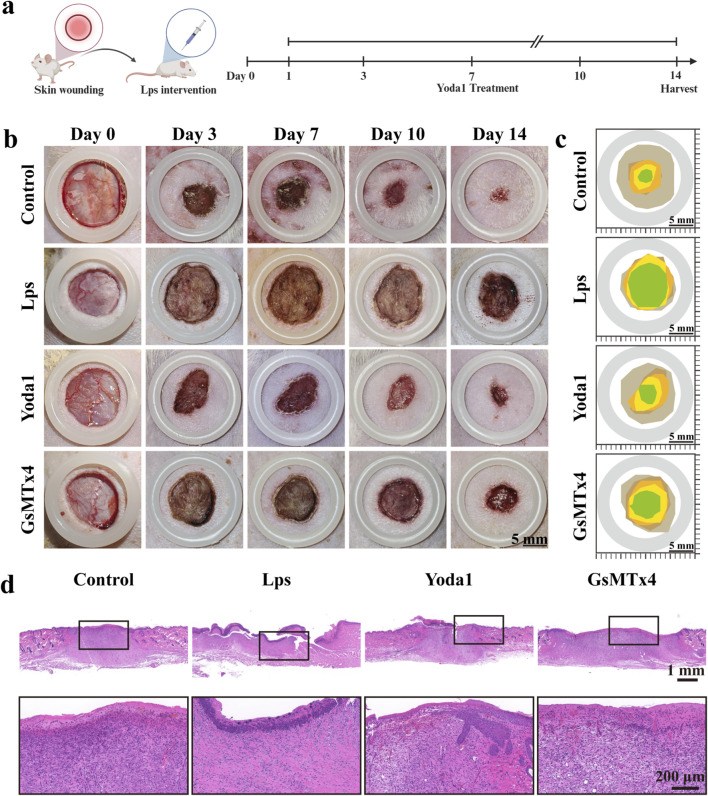
Yoda1 Enhances Skin Wound Healing in Rats. **(a)** Schematic diagram of the rat wound modeling procedure. **(b)** Representative photographs of wounds in different rat groups on days 0, 3, 7, 10, and 14 after wound modeling (n = 5 per group). **(c)** Overlay plot showing wound healing trajectories for different treatment groups, providing a visual comparison of wound closure speed and extent. **(d)** H&E staining of wound tissues collected on day 14 for evaluation. Low-magnification images display newly formed epithelial coverage in each group, while high-magnification images reveal morphological features of the new epithelium, including epithelial thickness, basal layer integrity, and development of skin appendages (n = 5 per group). All images were obtained from at least five rats. Data are presented as mean ± standard deviation (mean ± SD). Differences between groups were analyzed using one-way ANOVA (*P < 0.05, **P < 0.01, ***P < 0.001).

The wound modeling process and healing outcomes are illustrated in Figures 6a–c. Significant differences in wound closure rates were observed among the various treatment groups of rats. The normal control group exhibited the fastest wound healing, with the wound area rapidly decreasing over time, indicating a strong self-healing capacity under physiological conditions. In contrast, the LPS intervention group demonstrated the slowest wound healing rate, with a pronounced delay in wound closure, suggesting that inflammatory stimulation significantly impairs the wound repair process. Following Yoda1 treatment in conjunction with LPS, the wound healing rate significantly accelerated, and the wound closure rate was notably higher than that of the LPS group, indicating that Piezo1 activation effectively ameliorates the delayed healing phenotype induced by LPS. Conversely, combined treatment with GsMTx4 markedly diminished the wound healing improvement, further supporting that Yoda1’s pro-healing effect depends on the functional activation of Piezo1.

Hematoxylin and eosin (H&E) staining results are shown in Figure 6d. The normal control group exhibited an intact epidermal structure with good continuity and the highest degree of epithelialization. In the LPS group, the wound displayed a discontinuous epidermis and a thinner epithelial layer, accompanied by pronounced inflammatory cell infiltration, indicating impaired wound repair. Compared to the LPS group, the Yoda1 intervention group demonstrated significantly enhanced continuity of the newly formed epithelium, increased epithelial thickness, and a markedly improved degree of epithelialization, suggesting that Yoda1 effectively promotes the wound re-epithelialization process. In contrast, the GsMTx4 intervention group showed a significantly reduced degree of epithelialization, further supporting the critical role of Piezo1 in regulating the wound repair process.

### 3.7 Yoda1 activation of Piezo1 promotes collagen deposition, cell proliferation and angiogenesis

**FIGURE 7:**
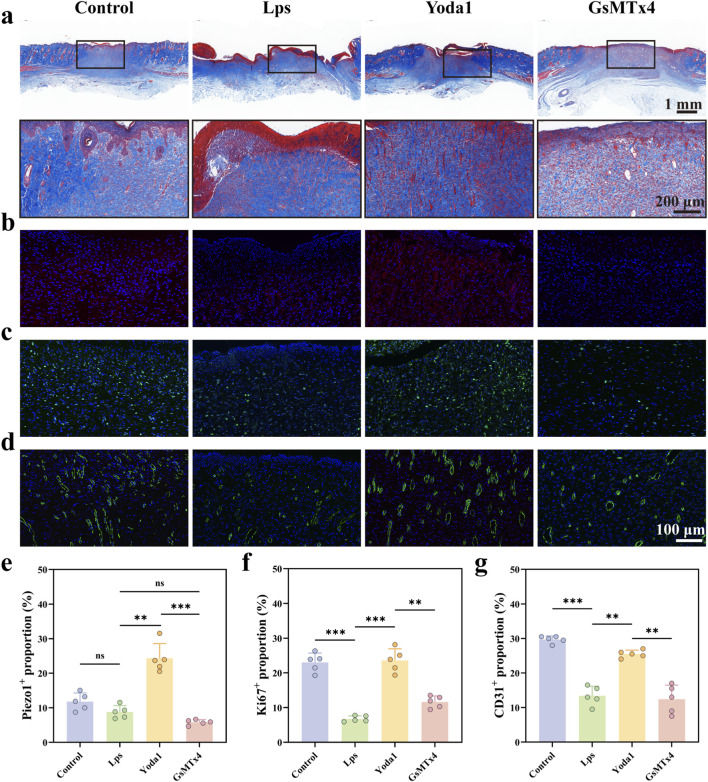
Yoda1 Promotes Collagen Deposition and Expression of Piezo1, Ki67, and CD31 in Rat Wound Tissue. **(a)** Masson’s trichrome staining of wound tissue on day 14. Low-magnification images show the coverage of newly formed epithelial tissue across different treatment groups, while high-magnification images display collagen fiber arrangement, thickness, and tissue structural integrity (n = 5 per group). **(b)** Immunofluorescence staining of Piezo1 activation (red) in wound tissues from various groups; nuclei were counterstained with DAPI (blue) (n = 5 per group). **(c)** Immunofluorescence staining of Ki67 (green) in wound tissues from different groups; nuclei were counterstained with DAPI (blue) to assess cell proliferation (n = 5 per group). **(d)** Immunofluorescence staining of CD31 (green) in wound tissues from different groups; nuclei were counterstained with DAPI (blue) to evaluate angiogenesis (n = 5 per group). **(e–g)** Quantitative analysis of the percentage of Piezo1-, Ki67-, and CD31-positive cells, respectively. Data are presented as mean ± standard deviation (mean ± SD), with at least five rats per group. Differences between groups were analyzed by one-way ANOVA (*P < 0.05, **P < 0.01, ***P < 0.001).

Masson’s trichrome staining results in Figure 7a show that the control group’s wound area exhibits a relatively regular and continuous distribution of collagen fibers, indicating a favorable tissue repair state. In contrast, the LPS-treated group displays a significant reduction in collagen deposition and a disorganized fiber arrangement, suggesting that inflammatory stimulation markedly inhibits the wound matrix repair process. Following Yoda1 intervention in the LPS-treated background, collagen deposition in the wound area significantly increases, and collagen fibers are more densely arranged, indicating that Piezo1 activation facilitates collagen remodeling and tissue regeneration. Conversely, the GsMTx4 intervention group shows a notable decrease in collagen deposition, suggesting that Yoda1’s pro-repair effects depend on the functional activation of Piezo1.

Immunofluorescence staining of Piezo1 is shown in Figures 7b,e. The Yoda1 intervention group exhibits a significant enhancement in Piezo1 fluorescence signal, indicating that Yoda1 effectively activates Piezo1 protein expression in the *in vivo* model. In contrast, the GsMTx4 intervention group shows a marked reduction in Piezo1 fluorescence intensity, demonstrating that Piezo1 activation is significantly inhibited by the inhibitor. Ki67 immunofluorescence staining was used to assess cell proliferation activity in the wound area (Figures 7c,f). The Yoda1 intervention group shows a significantly higher proportion of Ki67-positive cells in the wound area compared to the LPS group, indicating that Piezo1 activation can significantly enhance local cell proliferation capacity at the wound site. In contrast, the number of Ki67-positive cells was significantly reduced in both the LPS group and the GsMTx4 intervention group, indicating that inflammatory stimulation and Piezo1 inhibition are both detrimental to wound cell proliferation and repair.

CD31 immunofluorescence staining was used to assess angiogenesis and revascularization in the wound tissue. As shown in Figures 7d,g, the Yoda1 intervention group exhibited a significant increase in the number of CD31-positive blood vessels in the wound area, with a denser vascular distribution, suggesting that Piezo1 activation promotes tissue angiogenesis. In comparison, the LPS group and the GsMTx4 intervention group showed a significant decrease in the number of CD31-positive blood vessels, indicating that both inflammatory stimulation and Piezo1 inhibition markedly suppress the wound revascularization process. In summary, Yoda1 significantly promotes collagen deposition, cell proliferation, and angiogenesis by activating Piezo1, thereby improving LPS-induced wound healing impairment at multiple histological levels. This pro-repair effect is notably diminished when Piezo1 is inhibited by GsMTx4.

## 4 Discussion

Fibroblasts are the primary cell type in the skin of the maxillofacial region and the oral mucosa. Mechanical forces play a critical role in their homeostasis, migration, and matrix remodeling (Wang et al., 2025), while the mechanically sensitive ion channel Piezo1 is important in regulating cell fate (Rennekampff et al., 2024; Yang J. et al., 2025). This study found that Yoda1, a specific chemical agonist of Piezo1, exhibits a clear bidirectional effect on Piezo1 activation in fibroblasts. Low doses of Yoda1-induced Piezo1 activation synergistically enhance cell proliferation (Figure 1a), whereas higher concentrations have toxic effects (Lin et al., 2025). Previous studies have demonstrated that Piezo1 activation is closely related to cell proliferation in various cell types. Xing et al. found that Yoda1 activation of Piezo1 enhances the proliferation and osteogenic differentiation of human dental follicle cells (hDFCs) (Xing et al., 2022). Xue et al. reported that Yoda1 activation of Piezo1 promotes skin tissue growth by regulating mechanical signaling pathways, increasing proliferation markers in the skin matrix, and facilitating tissue expansion (Xue et al., 2025).

Furthermore, this study found that Yoda1 activation of Piezo1 enhances fibroblast migration, which helps restore collagen secretion impaired under inflammatory conditions and promotes fibroblast responses during tissue repair (Figure 2). Mechanistically, Yoda1 activation of Piezo1 triggers intracellular Ca^2+^ influx, which subsequently regulates the Hippo–YAP pathway, promoting YAP/TAZ nuclear translocation (Ma et al., 2025). The nuclear localization of YAP enhances cytoskeletal remodeling and cellular mechanical adaptability while meeting metabolic energy demands (Li X. et al., 2022; Liu et al., 2025), thereby significantly improving fibroblast migration and collagen secretion. Given Yoda1’s ability to enhance fibroblast viability and function in an inflammatory microenvironment, further investigation into the molecular mechanisms by which Yoda1 regulates fibroblast behavior in an LPS-induced inflammatory environment is essential to understanding the regulatory network involved in soft tissue repair.

**FIGURE 8:**
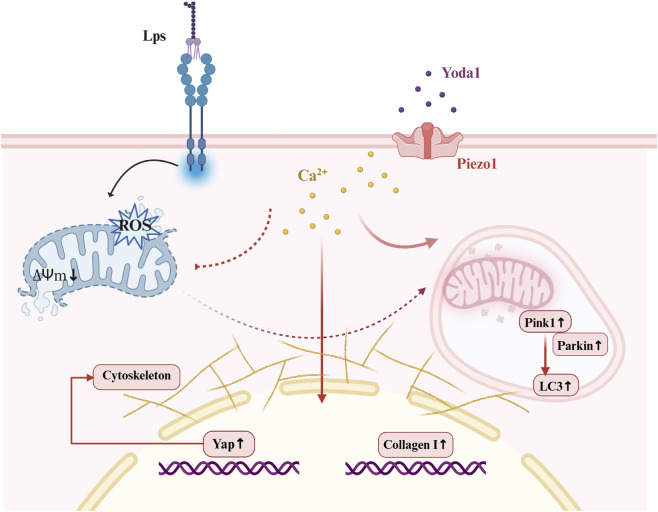
Illustrates the proposed mechanism by which Yoda1 modulates LPS-induced alterations in mitochondrial function and autophagy within cells.

Previous studies have confirmed that lipopolysaccharide (LPS), the primary endotoxin of Gram-negative bacteria, induces a significant oxidative stress response by activating Toll-like receptor-related signaling pathways. This activation inhibits the activity of respiratory chain complexes, leading to impaired ATP production (Alikhani et al., 2003). Moreover, accumulating evidence suggests that the influence of LPS on wound healing may exhibit dose-dependent characteristics, and the biological responses may also vary depending on the bacterial source of LPS (Cen et al., 2021), which could lead to distinct effects in oral tissues compared with facial skin. In this study, we observed that following LPS treatment, cellular reactive oxygen species (ROS) levels increased, accompanied by a decrease in mitochondrial membrane potential. These changes further triggered apoptosis and necrotic programmed cell death, severely impairing the biological functions of fibroblasts (Figures 4, 8). This suggests that disruption of mitochondrial homeostasis is a key pathological factor in LPS-induced cellular injury (Xu et al., 2025). Therefore, restoring mitochondrial homeostasis and preventing excessive ROS accumulation may be crucial strategies to mitigate inflammatory damage. Mitophagy, a critical mechanism for maintaining mitochondrial homeostasis, enhances cellular function by clearing damaged mitochondria, stabilizing membrane potential, and reducing oxidative stress. This mechanism has been widely reported across various cell types (Abulaiti et al., 2025).

In this study, we found that Yoda1 enhances mitophagy in fibroblasts by activating Piezo1, thereby maintaining mitochondrial homeostasis in an inflammatory microenvironment (Figure 5a). Furthermore, following Piezo1 activation, the expression levels of PINK1 and Parkin increased. The PINK1/Parkin pathway is a well-established signaling mechanism for selective mitophagy; accumulation of PINK1 on the outer mitochondrial membrane promotes the recruitment of Parkin and mediates the ubiquitination of damaged mitochondria, selectively clearing dysfunctional mitochondria to maintain cellular homeostasis (Lazarou et al., 2015; Wu et al., 2015). This pathway has been confirmed to activate in response to mitochondrial stress, facilitating the clearance of damaged mitochondria and preventing excessive reactive oxygen species (ROS) accumulation, thereby protecting cells from damage. Therefore, Piezo1 reduces ROS accumulation and maintains mitochondrial function by enhancing mitophagy, which in turn improves the proliferation and migration abilities of fibroblasts in an inflammatory environment (Figure 8).

Piezo1, as a mechanochemical transducer, can convert mechanical cues into intracellular Ca^2+^ signals, and the elevation of cytosolic calcium levels serves as a potential trigger for autophagy. Studies have shown that activation of Piezo1-mediated Ca^2+^ influx can regulate autophagic processes in neuronal cells through the Ca^2+^/calpain and calcineurin/TFEB pathways (Wu et al., 2025). Similarly, Xiao et al. reported that Piezo1-mediated Ca^2+^ influx enhances mitophagy in stem cells, thereby improving cellular recovery capacity (Xiao et al., 2025). This calcium-dependent mitophagy facilitates the efficient clearance of damaged mitochondria, reduces reactive oxygen species (ROS) accumulation, and maintains cellular homeostasis. Although Piezo1 acts as an upstream regulator of mitochondrial quality control, it is important to recognize that Ca^2+^ influx may elicit dual effects. On one hand, moderate Piezo1/Ca^2+^ signaling can activate protective pathways. On the other hand, excessive or sustained Ca^2+^ influx can lead to mitochondrial Ca^2+^ overload, ROS accumulation, and activation of pro-apoptotic pathways (Duchen, 2000).

Yoda1-activated Piezo1 enhances mitophagy and cell migration in human gingival fibroblasts, however, Piezo1 signaling may exert different biological effects depending on cell type and environmental context. It has been reported that activation of Piezo1 channels can promote mesenchymal stem cell (MSC) migration by inducing ATP release and activating P2 receptor-mediated purinergic signaling (Mousawi et al., 2020). In breast cancer, Piezo1 expression is upregulated, whereas inhibition of Piezo1 suppresses tumor cell migration (Yu et al., 2021). Yu et al. reported that Piezo1 affects the migration speed of LPS-treated C8-S astrocytes, revealing that astrocyte migration is significantly reduced in the presence of LPS (Yu et al., 2023). Although our results provide mechanistic insights for human gingival fibroblasts, caution should be exercised when extrapolating these findings to other cell types. Future studies should validate the broader applicability of these findings in multiple cell types involved in oral and maxillofacial tissue repair.

Soft tissue injury repair is a complex and highly coordinated physiological process. In the early stages of healing, fibroblasts migrate to the wound edges, proliferate, and synthesize extracellular matrix (ECM) to form granulation tissue and promote wound closure (Jiang et al., 2023). In the *in vivo* wound healing model used in this study, we observed that Yoda1-mediated activation of Piezo1 significantly accelerated wound closure, accompanied by enhanced deposition of type I collagen in the regenerating tissue at the wound edges (Figures 6, 7). During the proliferative phase, fibroblasts synthesize the primary ECM component, type I collagen, to support the structural stability of granulation tissue. Researchers found that Piezo1 plays a crucial role in mechanotransduction and ECM remodeling (He et al., 2024; Rashidi et al., 2025). As a mechanosensitive channel, Piezo1 integrates extracellular mechanical stimuli with cellular responses to ECM changes (Mousawi et al., 2020), thereby enhancing the overall efficiency of tissue regeneration (Zhu et al., 2023). Furthermore, angiogenesis is a critical component of the wound repair process. It provides pathways for oxygen, nutrients, and metabolic waste removal through endothelial cell sprouting and migration, meeting the high metabolic demands of cells during repair. Studies have shown that mechanical signals mediated by Piezo1 also influence the activity of pro-angiogenic pathways (Li M. et al., 2022), promoting the expression of vascular-related factors and maintaining the dynamic balance of neovascularization, thereby improving blood flow reperfusion and the repair environment (Chen et al., 2021; Kang et al., 2019). Under mechanical stimulation, Piezo1-mediated downstream signaling positively correlates with the expression of pro-angiogenic factors, which may explain the observed richer vascular network structures during tissue regeneration (Figure 7).

The ultimate goal of tissue repair is to restore cellular homeostasis and tissue function within complex environments. Imbalances in mitochondrial homeostasis and persistent oxidative stress are widely recognized as key pathological factors impairing the regeneration and repair of maxillofacial soft tissues (Pignet et al., 2024; Crompton et al., 2016). This study addresses this critical issue by proposing a novel strategy to regulate mitophagy and restore cellular function through Yoda1-mediated activation of the mechanosensitive ion channel Piezo1. We systematically elucidate the potential protective role of Yoda1 in alleviating LPS-induced mitochondrial damage and cell death. This finding not only provides new insights into the repair mechanisms of soft tissues under the combined influence of mechanical stimulation and microbial inflammatory signals but also enriches the theoretical framework of mechanobiology in the field of oral soft tissue regeneration. From a translational medicine perspective, the Piezo1-mediated mitophagy mechanism represents a promising therapeutic target for maxillofacial soft tissue regeneration (Xie et al., 2022). Future research should integrate local delivery strategies and tissue-specific regulatory approaches to validate therapeutic efficacy while ensuring biosafety, thereby advancing this mechanism from basic research toward clinical application.

## 5 Conclusion

This study demonstrates that activation of Piezo1 effectively mitigates LPS-induced damage in human gingival fibroblasts and promotes orofacial soft tissue wound repair. Piezo1 activation mediated by Yoda1 increases intracellular Ca^2+^ influx and facilitates YAP nuclear translocation, thereby enhancing fibroblast proliferation, migration, and type I collagen synthesis. Meanwhile, activited Piezo1 upregulates the PINK1/Parkin signaling pathway to promote mitophagy, which clears damaged mitochondria, reduces excessive ROS accumulation, and restores mitochondrial membrane potential, ultimately decreasing cell apoptosis. *In vivo* experiments further confirm that Piezo1 activation accelerates LPS-induced wound closure and promotes collagen deposition and angiogenesis. Collectively, Piezo1-mediated calcium regulation of mitophagy helps maintain fibroblast function under inflammatory conditions and facilitates soft tissue regeneration, providing a potential mechanobiological intervention target for maxillofacial wound healing.

## Data Availability

The original contributions presented in the study are included in the article/supplementary material, further inquiries can be directed to the corresponding authors.
